# Flexitarian Diets and Health: A Review of the Evidence-Based Literature

**DOI:** 10.3389/fnut.2016.00055

**Published:** 2017-01-06

**Authors:** Emma J. Derbyshire

**Affiliations:** ^1^Nutritional Insight Ltd., Surrey, UK

**Keywords:** flexitarian, semi-vegetarian diet, public health, gender differences, dietary trends

## Abstract

A flexitarian or semi-vegetarian diet (SVD) is one that is primarily vegetarian with the occasional inclusion of meat or fish. Of late, there appears to be an increasing movement toward this practice. There has not been a recent update on these diets from a health perspective. Using the National Centre for Biotechnology Information PubMed database, a search was made for all studies published between 2000 and 2016 that met defined inclusion criteria. A total of 25 studies were located with 12 focusing on body weight and diet quality. There was emerging evidence suggestive of benefits for body weight, improved markers of metabolic health, blood pressure, and reduced risk of type 2 diabetes. SVD may also have a role to play in the treatment of inflammatory bowel diseases, such as Crohn’s disease. Given that there is a higher tendency for females to be flexitarian yet males are more likely to overconsume meat, there is a clear need to communicate the potential health benefits of these diets to males.

## Introduction

“Flextarianism” is a neoteric term that has been emerging in the scientific and public sectors recently. Added to the Oxford English Dictionary in 2014, flexitarian is a portmanteau of “flexible” and “vegetarian,” referring to an individual who follows a primarily but not strictly vegetarian diet, occasionally eating meat or fish (1). Despite the global demands for meat, it appears that there are now a growing number of flexitarian consumers who abstain from eating meat regularly (2).

Most consumers can be grouped into meat consumers, meat avoiders, or meat reducers (3). The trend toward flexitarian diets (FDs) appears to reflect consumers who are “meat-reducers,” eating meat within meals on some but not every day of the week (3), as with typical “meat-eaters”. This definition is most closely in line with that of semi- or demi-vegetarianism. Subsequently, the terms are often used inter-changeably in the literature. For example, in one publication semi-vegetarian diets (SVDs) are defined as those significantly reducing meat intake on at least 3 days of the week (4).

The FD seems to recognize the fact that meat is an important source of protein, fat, and micronutrients (5, 6), yet also considers the ethical sides, such as the need to avoid intensification and improve animal welfare (7). It also considers evidence that long-term consumption of increasing amounts of red meat and particularly processed meat may increase the risk of mortality, cardiovascular disease, type 2 diabetes, and certain forms of cancer such as colon cancer (6). Recently, the International Agency for Research on Cancer classified red meat as probably carcinogenic and processed meat carcinogenic to humans (8).

Research from NatCen’s British Social Attitudes survey found that 29% of people in Britain have reduced the amount of meat that they ate in the past 12 months (9). The definition of meat reducers included reductions in all meats except fish. In particular, women (34%) were most likely to reduce their meat intake. Similarly, 39% of 65- to 79-year olds had reduced their red meat intake compared with 19% of 18- to 24-year olds. The report also showed that men (23%) were shifting and reducing their meat intake. Over half (58%) cited health reasons along with saving money, concerns over animal welfare, and food safety (9).

The aim of the present paper was to review the evidence looking at the FD/SVDs and health, using defined inclusion criteria. While some discussion papers have been published about vegetarianism (10) and low-meat diets and health (11), no publications have focused on the current trend of flexitarianism or SVDs. The present paper set out to evaluate the evidence-based looking as FD/SVDs from a public health perspective.

## Methods

The National Centre for Biotechnology Information (NCBI) search engine (PubMed) was used to extract relevant English-language papers published between January 2000 and June 2016. Data files were extracted from the NCBI collection depository and imported into Covidence software used to create systematic reviews.

As flexitarian is a relatively new term, the search terms “flexitarian,” “semi-vegetarian,” and “demi-vegetarian” were combined with “blood pressure,” “body weight,” “cancer,” “diabetes,” “diet quality,” “health,” “heart disease,” “metabolic health,” and “nutrient intake/status” to filter publications. Data extracted from each article included (1) country of research, (2) subjects (number of participants, gender, age), (3) design and methods, (4) definitions used, and (5) study outcomes/findings.

For inclusion, studies needed to clearly define the terms flexitarian, semi-vegetarian, or demi-vegetarian used in their study. Data results and findings also needed to be reported and analyzed separately from other forms of dietary patterns. Randomized controlled trials (RCTs) and observational studies were included. Articles were excluded if they were published before 2000, a pilot study, or focused solely on vegan diets or vegetarianism.

## Results

**Figure 1:**
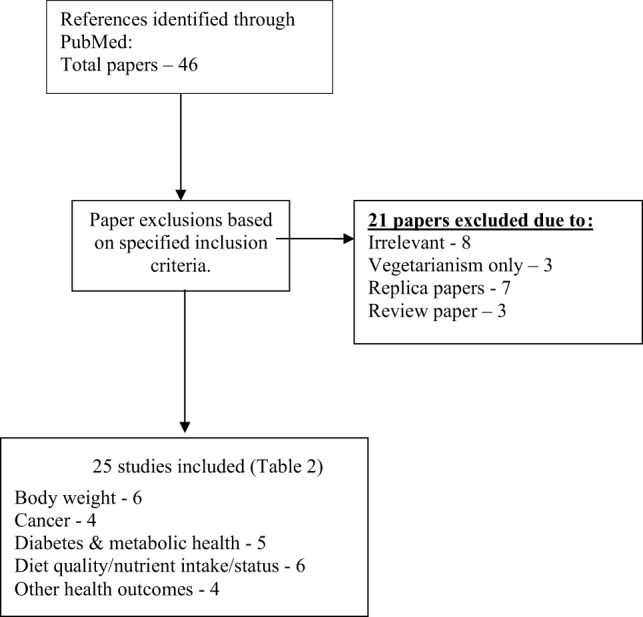
**Flow diagram for database search results**.

The NCBI search identified 46 papers and after an adjustment for replica papers, 39 articles remained for assessment. Of these, 14 papers were discarded after reviewing the abstracts and article content as they did not meet the inclusion criteria. This left 25 articles for general review. This included 21 epidemiological studies and 4 randomized controlled or clinical trials. The algorithm of qualifying publications is shown in Figure 1. Of these, 12 studies were conducted in the US or Canada, 6 in Europe, 5 in Asia, and 2 in Australasia.

### Definitions

Definitions of flexi-semi-vegetarianism were extracted from the identified papers (Table 1). Definitions tended to vary between studies. For example, some authors specified that participants on an SVD restricted their intake of red meat (12, 13), while others restricted fish (14). In studies using data from the Adventist Health Study-2, SVDs were defined as those consuming dairy products and/or eggs and meat (red meat and poultry ≥1 time/month and <1 time/week) (15–20).

**Table 1:** **Definitions of flexitarianism/semi-vegetarianism**.

Reference	Definition used
Baines et al. (13)	Excluded red meat but ate poultry and fish
Kornsteiner et al. (28)	Mostly lacto-vegetarian, sometimes eating fish and poultry or eggs
Tonstad et al. (15)	Consumed dairy products and/or eggs and meat (red meat and poultry ≥1 time/month and <1 time/week)
Chiba et al. (29)	Miso (fermented bean paste) soup, vegetables, fruits, legumes, potatoes, pickled vegetables, and plain yogurt were served daily. Fish was served once a week and meat once every 2 weeks, both at about a half the average amount
Rizzo et al. (17)	Consumed fish at any frequency but consumed other meats <1 time/month or total meat (with red meat and poultry ≥1 time/month and <1 time/week)
Rodenas et al. (27)	Eat certain foods of animal origin but usually exclude red meat from their diet
Orlich et al. (19)	Consumed non-fish meats 1 or more times/month and all meats combined (fish included) 1 or more times/month but 1 or less time/week
Rizzo et al. (18)	Consumed dairy products and/or eggs, ate some meat (red meat and poultry) ≥1 time/month, and the total of fish and meat ≥1 time/month but <1 time/week
Tantamango-Bartley et al. (26)	Ate red meat, poultry, fish 1/month to 1/week, and eggs or dairy at any level
Tonstad et al. (16)	Consumed dairy products and/or eggs and (red meat and poultry ≥1 time/month and <1 time/week)
Agrawal et al. (14)	Consumed fruits, vegetables, pulses or beans, and animal products (chicken or meat, eggs, milk, or curd) either daily, weekly, or occasionally but no fish
Clarys et al. (23)	Consuming red meat, poultry, or fish no more than once a week
Chiba et al. (30)	A lacto-ovo-vegetarian diet with an additional serving of fish once a week and meat once every 2 weeks
Kim and Bae (12)	Had restricted the intake of meat and some food groups for at least 20 years
Moore et al. (21)	Diets recommending moderate levels of animal intake
Orlich et al. (20)	Consumed non-fish meats 1 or more times/month and all meats combined (fish included) 1 or more times/month but 1 or less time/week
Turner-McGrievy et al. (22)	Diets recommending moderate levels of animal intake
Penniecook-Sawyers et al. (24)	Intake of red meats, poultry, or fish, but not only fish was more than or equal to once per month but less than once per week
Tantamango-Bartley et al. (25)	Ate a total of red meat or poultry ≥1 time/month but all meats combined (including fish) <1 time/week and eggs/dairy in any amount

In a more general sense, other authors defined SVDs as those containing moderate levels of animal products (21, 22), though it was not specified what “moderate” was. One paper defined these as eating red meat, poultry, or fish no more than once a week (23), while other studies reported participants as being semi-vegetarian (SV) if they excluded red meat from their diet but ate other meats (13).

## Health

### Body Weight

As shown in Table 2, six studies focused on SVDs and body weight. Two RCTs looked at the effects of different plant-based diets in relation to weight loss. In one study, authors undertook a 6-month RCT, where overweight adults were allocated to five different plant-based diets. Vegan diets were associated with significantly higher levels of weight loss by the end of the study (22). A Korean study reported that postmenopausal women maintaining an SVD over 20 years had a significantly lower body weight, body mass index (BMI), and percentage of body fat compared with non-vegetarians (NVs) (12).

**Table 2:** **Information extracted from studies looking at flexitarianism in relation to diet quality and aspects of health**.

Reference; country	Subjects	Study design and methods	Findings
**Body weight**			

Kim and Bae (12); Korea	Postmenopausal SV F (*n* = 54). Mean age 61.4 years	Cross-sectional. Anthropometric and blood biomarkers compared between the two groups	SV had sig. lower body weight (*P* < 0.01), BMI (*P* < 0.001), and percentage of body fat (*P* < 0.001) than the NV

Moore et al. (21); US	*n* = 15 SV/PVs	6-month RCT. New DIETs study—randomization to one of four plant-based diets (vegan, vegetarian, PV, SV) or an omnivore diet	At 6 months, non-adherent vegan and vegetarian participants had a sig. decrease in cholesterol intake than non-adherent PV/SV (*P* = 0.02) or omnivore participants (*P* = 0.04)

Turner-McGrievy et al. (22); US	*n* = 13 SV (18–65 years)	6-month RCT. Overweight and obese adults randomized to different diets for 6 months	At 6 months, the weight loss in the vegan group (−7.5%) was higher than the omnivorous (−3.1%; *P* = 0.03), SV (−3.2%; *P* = 0.03), and PV (−3.2%; *P* = 0.03) groups

Rizzo et al. (18); US and Canada	*n* = 4,042. Mean age 33.3 years. 67% F)	Cross-sectional. Data analyzed from Adventist Health Study-2. 204-item FFQ used to compare nutrient profiles	NV had the highest mean BMI values (28.7) and highest proportion of obese subjects (33.3%) when compared to any other dietary pattern

Tonstad et al. (15); US	*n* = 3,386 SV. Mean age 57.7 years. 65.7% F	Cross-sectional. Analysis of different types of vegetarian diet from the Adventist Health Study-2. Anthropometric data collected from a self-administered questionnaire	Mean BMI was lowest in vegans (23.6 kg/m^2^) and incrementally higher in LOV (25.7 kg/m^2^), PV (26.3 kg/m^2^), SV (27.3 kg/m^2^), and NV (28.8 kg/m^2^)

Baines et al. (13); Australia	*n* = 827 SV F (22–27 years)	Cross-sectional. Data analyzed from the Australian Longitudinal Study on Women’s Health	SV had a lower BMI (mean 23 kg/m^2^ than NVs (23.7 kg/m^2^) and tended to exercise more

**Cancer**			

Tantamango-Bartley et al. (25); US	*n* = 26,346 M	Prospective cohort study. Data analyzed from Adventist Health Study-2	Vegan diets showed a statistically sig. protective association with prostate cancer risk (HR: 0.65; 95% CI: 0.49, 0.85)

Penniecook-Sawyers et al. (24); US	*n* = 2,930 SV F	7.8-year Prospective cohort. Data analyzed from Adventist Health Study-2. FFQ used to group diets and BC incidence measured	There was no evidence that vegetarians as a group had lower risk of BC than NVs either in pre- or postmenopausal, or in Black or White, women

Orlich et al. (20); US	*n* = 4,271. 67.8% F. 58.6 years	7.3-year Prospective cohort. Data analyzed from Adventist Health Study-2. FFQ used to group diets and cases of colorectal cancer identified	Adjusted HR in vegans was 0.84; in LOV, 0.82; in PV, 0.57; and in SV 0.92 compared with NV. Mean fiber intake in SV was 30.4 g/day and calcium intakes 821 mg/day

Tantamango-Bartley et al. (26); US	*n* = 3,881 69% F	4.1-year Prospective cohort. Data analyzed from Adventist Health Study-2	Vegan diets showed statistically sig. protection for overall cancer incidence (HR = 0.84) in both genders combined and for female-specific cancers (HR = 0.66). LOV were associated with decreased risk of cancers of the GI system

**Diabetes and metabolic health**			

Kim and Bae (12); Korea	Postmenopausal SV F (*n* = 54). Mean age 61.4 years	Cross-sectional. Blood biomarkers compared between the two groups	SV had sig. lower body weight (*P* < 0.01), BMI (*P* < 0.001), % of body fat (*P* < 0.001), and serum levels of leptin (*P* < 0.05), glucose (*P* < 0.001), and insulin (*P* < 0.01), than the NV

Agrawal et al. (14); India	*n* = 8,140 SV, *n* = 4,675 F (20–49 years)	Cross-sectional. Data from India’s third National Family Health Survey (2005–2006)	Consumption of a lacto- (OR: 0.67, *P* < 0.01), LOV (OR: *P* = 0.03), and SV (OR: *P* = 0.03) diet was associated with a lower likelihood of diabetes than an NV diet in the adjusted analyses

Tonstad et al. (16); US	*n* = 2,404, 65.7% F	2-year Prospective cohort. Analysis of different types of vegetarian diet and lifestyle data from the Adventist Health Study-2	In non-Blacks vegan, lacto ovo and SV diets were protective against diabetes (OR 0.429, OR 0.684, OR 0.501)

Rizzo et al. (17); US	*n* = 773 subjects; 16% SV (mean age 60 years)	Cross-sectional. Data analyzed from Adventist Health Study-2. Fasting blood samples taken and WC measured	MetS was highest in NV (39.7%), intermediate in SV (37.6%), and lowest in vegetarians (25.2%)

Rodenas et al. (27); Spain	*n* = 14 SV F	Cross-sectional. Blood pressure of postmenopausal F measured	Omnivores had sig. higher systolic (*P* < 0.01) and diastolic (*P* < 0.05) pressures than SVs

Tonstad et al. (15); US	*n* = 3,386 SV. Mean age 57.7 years. 65.7% F	Cross-sectional. Analysis of different types of vegetarian diet from the Adventist Health Study-2. Blood samples provided by a sub-sample	Prevalence of T2D increased from 2.9% in vegans to 7.6% in NV; the prevalence was intermediate in participants consuming LOV (3.2%), pesco (4.8%), or SV (6.1%) diets

**Diet quality**			

Turner-McGrievy et al. (22); US	*n* = 13 SV	6-month RCT. Overweight and obese adults randomized to five different plant-based diets.	Vegan, vegetarian, and PV subjects had sig. improvements in the dietary inflammation index score compared with SV participants at 2 months (*P* < 0.05) with no differences at 6 months

Clarys et al. (23); Belgium	*n* = 498 SV (20–69 years)	Cross-sectional. Online 52-item FFQ completed	SVs had some of the highest calcium intakes (1,470 mg/day) and had one of the strongest nutrient densities

Rizzo et al. (18); US and Canada	*n* = 4,042 SV. 67.3% F. Mean age 33.3 years.	Cross-sectional study. Data from the Adventist Health Study-2. 204-item validated semi-quantitative FFQ	SV had an intake of 1,713 kcal/day and the lowest energy intakes

Rodenas et al. (27); Spain	*n* = 14 SV F	Cross-sectional. 14-day weighing of foods to identify mineral intakes	The omnivorous diet contained a significantly higher mineral content than the SV one

Kornsteiner et al. (28); Austria	*n* = 13 SV	Observational study. Amount and composition of ingested fat were calculated from 24-h recalls	The unbalanced *n*-6/*n*-3 ratio and the limited dietary sources of EPA and DHA in vegans and vegetarians led to reductions in C20:5n-3, C22:5n-3, C22:6n-3, and total *n*-3 fatty acids compared with omnivores and semi-omnivores

Baines et al. (13); Australia	9,113 F (22–27 years)	Cross-sectional. Data analyzed from the Australian Longitudinal Study on Women’s Health	The BMI and levels of physical activity of SV women suggest they were healthier than NV. There were greater reports of menstrual problems and the poorer mental health which could be of clinical significance

**Other health outcomes**			

Chiba et al. (30); Japan	*n* = 22 adults patients with CD	Clinical Trial. Initiated a high-fiber SV diet containing 32.4 g fiber per 2,000 kcal among patients in remission with CD	High-fiber SV diets may have a role to play in the treatment of CD

Chiba et al. (29); Japan	*n* = 22 adults patients with CD	Prospective, single center, 2-year clinical trial was conducted. Patients in clinical remission were advised to continue with an SVD and avoid known high-risk foods for IBD	The concentration of C-reactive protein was normal at the final visit in more than half of the patients in remission who were on the SV diet. There was no untoward effect of SV diet

Orlich et al. (19); US	*n* = 4,031 SV	5.9-year Prospective cohort. Data analyzed from Adventist Health Study-2	The adjusted HR for all-cause mortality in vegans was 0.85; in LOVs, 0.91; in PV 0.81; and in SV, 0.92 compared with NV

*BC, breast cancer; BMI, body mass index; CD, Crohn’s disease; CI, confidence interval; DHA, docosahexaenoic acid; EPA, eicosapentaenoic acid; F, female; FFQ, food frequency questionnaire; HR, hazard ratio; IBD, inflammatory bowel disease; LOV, lacto-ovo-vegetarian; M, Male; MetS, metabolic syndrome; NV, non-vegetarian; OR, odds ratio; PV, pesco-vegetarian; RCT, randomized controlled trial; sig, significantly; SV, semi-vegetarian; SVD, semi-vegetarian diet; T2D, type 2 diabetes; WC, waist circumference*.

Cross-sectional data from 71,751 participants taking part in the Adventist Health Study-2 (2002–2007) showed that BMI was highest in NVs (mean 28.7 kg/m^2^), slightly lower in SVs (mean 27.4 kg/m^2^), and lowest in strict vegetarians (mean 24.0 kg/m^2^) (18). These findings are similar to earlier trends (2002–2006 analysis) showing that mean BMI was lowest in vegans (23.6 kg/m^2^) and incrementally higher in lacto-ovo vegetarians (LOVs) (25.7 kg/m^2^), pesco-vegetarians (PVs) (26.3 kg/m^2^), SVs (27.3 kg/m^2^), and NVs (28.8 kg/m^2^) (15). Cross-sectional research on 9,113 young Australian women (22–27 years) identified that SVs had a lower BMI and tended to exercise more than NVs (13).

### Cancer

Four studies were found to fulfill the inclusion criteria. One study examined vegetarian patterns in relation to breast cancer (BC) risk using data from 96,001 adults taking part in the prospective Adventist Health Study-2 (2002–2007). Findings showed that vegans had a significantly lower risk of BC compared with vegetarian and NVs (24).

With regard to prostate cancer risk, data from 26,346 males taking part in the Adventist Health Study-2 found that only vegan diets were associated with reduced prostate cancer risk (25). Equally, an earlier analysis of this study showed that vegan diets had statistically significant protection for overall cancer incidence (hazard ratio = 0.84; 95% confidence interval: 0.72, 0.99) (26). Other work using data from the same North American study discovered that PVs had the lowest risk of colorectal cancer, followed by LOVs, vegans, and SVs when compared with NVs (20).

### Diabetes and Metabolic Syndrome

Six studies were found looking at SVDs in relation to markers of metabolic health or risk of diabetes. In one study, authors observed that postmenopausal women following an SVD for more than 20 years had significantly lower glucose, insulin levels, and homeostatic model assessment of insulin resistance compared with NV controls (12).

Other research using data from India’s third National Family Health Survey 2005–2006 (*n* = 156,317) from adults aged 20–49 years found that the consumption of lacto-, lacto-ovo-, and SVDs were associated with a reduced likelihood of diabetes compared with NV diets, after data adjustments (14). Equally, research from the Adventist Health Study-2 found that cases of diabetes developed were lowest in vegans (0.54%), followed by SVs (0.92%), LOVs (1.1%), and PVs (1.3%) compared with 2.1% in NVs (16).

Furthermore, cross-sectional data from the Adventist Health Study-2 (*n* = 773) showed that the prevalence of metabolic syndrome was highest in NVs (39.7%), intermediate in SVs (37.6%), and lowest in vegetarians (25.2%). Data for vegans were not reported (17). Findings from the same study also highlighted that diabetes prevalence was 2.9% in vegans, 4.8% in SVs, and 7.6% in NVs (15). With regard to blood pressure, research involving 26 postmenopausal women from convents found that omnivores had significantly higher systolic and diastolic pressures than the SVs (27).

### Diet Quality

Six studies focused on dietary quality, nutritional intakes, and/or status. In a randomized trial, overweight and obese adults were allocated to different plant-based diets. Participants allocated to the vegan and vegetarian diets had significantly improved macronutrient profiles and Diet Inflammatory Index (DII) scores (a tool for assessing the inflammatory potential of a diet). Vegan, vegetarian, and PV groups all had significant improvement in the DII score at 2 months but not at 6 months (22).

Remaining studies were mainly observational in nature. For example, an internet-based survey using data collection tools from the Belgian Food Consumption Survey and a convenience sample of subjects showed that vegan diets had the lowest total energy and highest fiber intake compared with omnivores (23). Alongside this, results from 96,335 adults in the Adventist Health Study-2 showed found that those eating SVDs had the lowest caloric intakes (1,713 kcal/day) but other than this there were few dietary differences (18).

Cross-sectional research from the Australian Longitudinal Study on Women’s Health (*n* = 9,113) showed that rates of low iron, iron-deficiency, or anemia were highest in vegetarians (42.6%), followed by SVs (38.6%) and then NVs (25.5%) (13). Other research focusing on the omega-3 profile of diets showed that vegan and vegetarian diets led to reductions in eicosapentaenoic acid and docosahexaenoic acid levels compared with semi-omnivores (28).

### Additional Health Outcomes

Three studies focused on other health outcomes. One study observed that SVDs helped to prevent relapse of symptoms in patients with inflammatory bowel disease (IBD) (29). Additional research by the same team concluded that up to 32.4 g dietary fiber, delivered *via* an SVD could be given to patients with IBD, indicating that these could be used as a supportive treatment for Crohn’s disease patients (30).

Other work involving 73,308 adults from the Seventh-day Adventist study-2 demonstrated that vegetarian diets, including SVDs were associated with lower all-cause mortality, with results appearing to be more robust in males (19).

## Discussion

Flexitarian diets have been gaining popularity—a transition that seems to have been fueled by a combination of health, environmental, and animal welfare concerns. The present paper has identified that flexitarian/SVDs could have potential health benefits with strongest evidence appearing to be in relation to weight loss and metabolic health benefits, including reduced diabetes risk and blood pressure (Table 2).

There is also emerging evidence that SVDs could be an option for patients with IBD, such as Crohn’s disease (30). While the mechanisms behind this are yet to be confirmed, it has been speculated that a plant-based diet may be effective for gut inflammation, namely, through the actions of dietary fiber (31).

Physicians focused on non-invasive and cost-effective interventions could help their patients improve health outcomes by encouraging a shift toward diets higher in vegetables, whole grains, legumes and fruits, and fewer animal products (32) with an FD being a useful tool in this transition. Furthermore, it has been estimated that making a transition toward plant-based diets that are aligned with standard dietary guidelines could help to reduce global mortality by 6–10% (33).

Flexitarian diets/semi-vegetarian diets could also be useful in helping those with high meat intakes to fall in line with recommended guidelines. For example, data from the UK National Diet and Nutrition Survey showed that red and processed meat intakes were 84 g/day for men and 47 g/day for women (34). Scientific Advisory Committee on Nutrition guidelines are set at 70 g/day for adults for red and processed meat (35). Subsequently, there is a greater tendency for males to exceed red and processed meat guidelines. Interestingly, looking at the studies reviewed, around 70% of conscious flexitarians were educated females (15, 18, 20, 26). Considering this, there is clear scope to educate males about the health benefits of FDs.

In order to align with meat intake guidelines other approaches can also be taken. These include a greater use of simulated meat-like products with a similar flavor, texture, and color to meat (36). As it is the International Year of the Pulse, there has been much interest in these from a health perspective. Pulses include beans, peas, and lentils, which have been eaten for at least 10,000 years, providing protein, fiber, and essential micronutrients, including iron, folate zinc, magnesium, as well as phytochemicals, such as saponins and tannins (37). It has been estimated that eating just half a cup of beans or peas daily can significantly enhance diet quality and nutrient density, helping consumers to meet dietary recommendations (37).

### Limitations

On the whole, FDs appear to have emerging health benefits. However, it should be considered that before any formal recommendations about FDs can be made, official definitions of these diets are needed. For example, the inclusion criteria used in the present article did not always bring up publications using the same definition of SVDs. In Germany, legal definitions of vegetarian and vegan have been compiled by the German Federation of Food Law and Food Science and the Vegetarierbund Deutschland to facilitate the categorizing and labeling of foods, though these definitions are yet to be formalized by the European Commission (38). Similarly, a formal classification of FDs is needed that can be put into appropriate use in future research.

Presently, large cohort investigations also appear to overlook FD/SVDs. For example, the recent European Prospective Investigation into Cancer and Nutrition did not include SVDs when comparing the metabolic profile of meat eaters, vegetarians, and vegans (39). It is also worth pointing out that large dietary surveys, such as the UK National Diet and Nutrition Survey, could look to include a definition and analysis of flexitarian or SVDs.

Equally, impending RCTs looking to investigate the health benefits of FD/SVDs need to align methodologies with the Consolidated Standards of Reporting Trials guidelines for RCTs (40). In the case of observational studies, these should follow the Strengthening the Reporting of Observational studies in Epidemiology guidelines (41). It was a limitation in the present review that the most research came from the US and Canadian Adventist Health Study. The SV dietary approaches taken by these Adventists may be different that of other flexitarians or SVs. More research in the UK and Europe is needed to examine this.

One a final note, in the present review, the focus was on “flexitarianism” as this terminology has been increasingly used by the public press. Nevertheless, it should be considered that terms such as “meat reduction” or “Mediterranean diets” which may also constitute an FD were not included in this review. These could form the basis of future publications, along with an anticipated future growth in studies looking at FD and markers of health.

## Conclusion

The trend of flexitarianism does not appear to be subsiding. This review provides a first line of evidence that FDs may have emerging health benefits in relation to weight loss, metabolic health, and diabetes prevention. While most flexitarians presently seem to be female, there is a clear need to communicate the potential health benefits of these diets to males. As not everyone and in particular men might not want to exclude meat altogether, FDs offer a path that includes their dietary preferences yet could improve public health outcomes.

## Author Contributions

This review has been researched and written by an Independent Nutrition Consultant.

## Conflict of Interest Statement

The author declares that the research was conducted in the absence of any commercial or financial relationships that could be construed as a potential conflict of interest. The reviewer AT and handling Editor declared their shared affiliation, and the handling Editor states that the process nevertheless met the standards of a fair and objective review.

## References

[1] Oxford English Dictionary. The Definitive Record of the English Language. (2014). Available from: http://www.oed.com

[2] Dagevos H Flexibility in the frequency of meat consumption – empirical evidence from the Netherlands. EuroChoices (2014) 13(2):40–5. 10.1111/1746-692X.12062

[3] Dagevos H Voordouw J Sustainability and meat consumption: is reduction realistic? Sustainability Sci Pract Policy (2013) 9(2):60–9.

[4] De Backer CJ Hudders L. From meatless Mondays to meatless Sundays: motivations for meat reduction among vegetarians and semi-vegetarians who mildly or significantly reduce their meat intake. Ecol Food Nutr (2014) 53(6):639–57. 10.1080/03670244.2014.896797 25357269

[5] Pighin D Pazos A Chamorro V Paschetta F Cunzolo S Godoy F A contribution of beef to human health: a review of the role of the animal production systems. Scientific World Journal (2016). 10.1155/2016/8681491 26989765 PMC4771914

[6] Battaglia Richi E Baumer B Conrad B Darioli R Schmid A Keller U. Health risks associated with meat consumption: a review of epidemiological studies. Int J Vitam Nutr Res (2015) 85(1–2):70–8. 10.1024/0300-9831/a000224 26780279

[7] Goldberg AM Farm animal welfare and human health. Curr Environ Health Rep (2016) 3(3):313–21. 10.1007/s40572-016-0097-9 27344143

[8] IARC Monographs Evaluate Consumption of Red Meat and Processed Meat. (2015). Available at: https://www.iarc.fr/en/media-centre/pr/2015/pdfs/pr240_E.pdf

[9] NatCen. Are We Eating Less Meat? A British Social Attitudes Report. (2016). Available at: http://www.natcen.ac.uk/our-research/research/british-social-attitudes-are-we-eating-less-meat

[10] Pilis W Stec K Zych M Pilis A. Health benefits and risk associated with adopting a vegetarian diet. Rocz Panstw Zakl Hig (2014) 65(1):9–14. 24964573

[11] McEvoy CT Temple N Woodside JV. Vegetarian diets, low-meat diets and health: a review. Public Health Nutr (2012) 15(12):2287–94. 10.1017/S1368980012000936 22717188 PMC10271837

[12] Kim MH Bae YJ. Comparative study of serum leptin and insulin resistance levels between Korean postmenopausal vegetarian and non-vegetarian women. Clin Nutr Res (2015) 4(3):175–81. 10.7762/cnr.2015.4.3.175 26251836 PMC4525134

[13] Baines S Powers J Brown WJ. How does the health and well-being of young Australian vegetarian and semi-vegetarian women compare with non-vegetarians? Public Health Nutr (2007) 10(5):436–42. 10.1017/S1368980007217938 17411462

[14] Agrawal S Millett CJ Dhillon PK Subramanian SV Ebrahim S. Type of vegetarian diet, obesity and diabetes in adult Indian population. Nutr J (2014) 13:89. 10.1186/1475-2891-13-89 25192735 PMC4168165

[15] Tonstad S Butler T Yan R Fraser GE. Type of vegetarian diet, body weight, and prevalence of type 2 diabetes. Diabetes Care (2009) 32(5):791–6. 10.2337/dc08-1886 19351712 PMC2671114

[16] Tonstad S Stewart K Oda K Batech M Herring RP Fraser GE. Vegetarian diets and incidence of diabetes in the adventist health study-2. Nutr Metab Cardiovasc Dis (2013) 23(4):292–9. 10.1016/j.numecd.2011.07.004 21983060 PMC3638849

[17] Rizzo NS Sabate J Jaceldo-Siegl K Fraser GE. Vegetarian dietary patterns are associated with a lower risk of metabolic syndrome: the adventist health study 2. Diabetes Care (2011) 34(5):1225–7. 10.2337/dc10-1221 21411506 PMC3114510

[18] Rizzo NS Jaceldo-Siegl K Sabate J Fraser GE. Nutrient profiles of vegetarian and nonvegetarian dietary patterns. J Acad Nutr Diet (2013) 113(12):1610–9. 10.1016/j.jand.2013.06.349 23988511 PMC4081456

[19] Orlich MJ Singh PN Sabate J Jaceldo-Siegl K Fan J Knutsen S Vegetarian dietary patterns and mortality in adventist health study 2. JAMA Intern Med (2013) 173(13):1230–8. 10.1001/jamainternmed.2013.6473 23836264 PMC4191896

[20] Orlich MJ Singh PN Sabate J Fan J Sveen L Bennett H Vegetarian dietary patterns and the risk of colorectal cancers. JAMA Intern Med (2015) 175(5):767–76. 10.1001/jamainternmed.2015.59 25751512 PMC4420687

[21] Moore WJ McGrievy ME Turner-McGrievy GM. Dietary adherence and acceptability of five different diets, including vegan and vegetarian diets, for weight loss: the new DIETs study. Eat Behav (2015) 19:33–8. 10.1016/j.eatbeh.2015.06.011 26164391

[22] Turner-McGrievy GM Wirth MD Shivappa N Wingard EE Fayad R Wilcox S Randomization to plant-based dietary approaches leads to larger short-term improvements in dietary inflammatory index scores and macronutrient intake compared with diets that contain meat. Nutr Res (2015) 35(2):97–106. 10.1016/j.nutres.2014.11.007 25532675

[23] Clarys P Deliens T Huybrechts I Deriemaeker P Vanaelst B De Keyzer W Comparison of nutritional quality of the vegan, vegetarian, semi-vegetarian, pesco-vegetarian and omnivorous diet. Nutrients (2014) 6(3):1318–32. 10.3390/nu6031318 24667136 PMC3967195

[24] Penniecook-Sawyers JA Jaceldo-Siegl K Fan J Beeson L Knutsen S Herring P Vegetarian dietary patterns and the risk of breast cancer in a low-risk population. Br J Nutr (2016) 115(10):1790–7. 10.1017/S0007114516000751 26987270 PMC4907539

[25] Tantamango-Bartley Y Knutsen SF Knutsen R Jacobsen BK Fan J Beeson WL Are strict vegetarians protected against prostate cancer? Am J Clin Nutr (2016) 103(1):153–60. 10.3945/ajcn.114.106450 26561618 PMC4691666

[26] Tantamango-Bartley Y Jaceldo-Siegl K Fan J Fraser G. Vegetarian diets and the incidence of cancer in a low-risk population. Cancer Epidemiol Biomarkers Prev (2013) 22(2):286–94. 10.1158/1055-9965.EPI-12-1060 23169929 PMC3565018

[27] Rodenas S Sanchez-Muniz FJ Bastida S Sevillano MI Larrea Marin T Gonzalez-Munoz MJ. Blood pressure of omnivorous and semi-vegetarian postmenopausal women and their relationship with dietary and hair concentrations of essential and toxic metals. Nutr Hosp (2011) 26(4):874–83. 10.1590/S0212-16112011000400030 22470037

[28] Kornsteiner M Singer I Elmadfa I. Very low n-3 long-chain polyunsaturated fatty acid status in Austrian vegetarians and vegans. Ann Nutr Metab (2008) 52(1):37–47. 10.1159/000118629 18305382

[29] Chiba M Abe T Tsuda H Sugawara T Tsuda S Tozawa H Lifestyle-related disease in Crohn’s disease: relapse prevention by a semi-vegetarian diet. World J Gastroenterol (2010) 16(20):2484–95. 10.3748/wjg.v16.i20.2484 20503448 PMC2877178

[30] Chiba M Tsuji T Nakane K Komatsu M. High amount of dietary fiber not harmful but favorable for Crohn disease. Perm J (2015) 19(1):58–61. 10.7812/TPP/14-124 25663207 PMC4315379

[31] Chiba M Ohno H Ishii H Komatsu M Plant-based diets in Crohn’s disease. Perm J (2014) 18(4):94 10.7812/TPP/14-117 PMC4315368 25662787

[32] Tuso PJ Ismail MH Ha BP Bartolotto C. Nutritional update for physicians: plant-based diets. Perm J (2013) 17(2):61–6. 10.7812/TPP/12-085 23704846 PMC3662288

[33] Springmann M Godfray HC Rayner M Scarborough P. Analysis and valuation of the health and climate change cobenefits of dietary change. Proc Natl Acad Sci U S A (2016) 113(15):4146–51. 10.1073/pnas.1523119113 27001851 PMC4839446

[34] Bates B Lennox A Prentice A Bates C Page P Nicholson S National Diet and Nutrition Survey Results from Years 1, 2, 3 and 4 (Combined) of the Rolling Programme (2008/2009 – 2011/2012). (2016). Available from: http://www.gov.uk/government/statistics/national-diet-and-nutrition-survey-results-from-years-1-to-4-combined-of-the-rolling-programme-for-2008-and-2009-to-2011-and-2012

[35] Scientific Advisory Committee on Nutrition. Iron and Health. (2010). Available from: http://www.gov.uk/government/publications/sacn-iron-and-health-report

[36] Kumar P Chatli MK Mehta N Singh P Malav OP Verma AK. Meat analogues: health promising sustainable meat substitutes. Crit Rev Food Sci Nutr (2015). 10.1080/10408398.2014.939739 25898027

[37] Mudryj AN Yu N Aukema HM. Nutritional and health benefits of pulses. Appl Physiol Nutr Metab (2014) 39(11):1197–204. 10.1139/apnm-2013-0557 25061763

[38] Michali N Germany Back Proposal for Legal Definition of Vegetarian and Vegan Food. (2016). Available from: http://www.foodnavigator.com/Policy/Germany-backs-proposal-for-legal-definition-of-vegetarian-and-vegan-food

[39] Schmidt JA Rinaldi S Ferrari P Carayol M Achaintre D Scalbert A Metabolic profiles of male meat eaters, fish eaters, vegetarians, and vegans from the EPIC-Oxford cohort. Am J Clin Nutr (2015) 102(6):1518–26. 10.3945/ajcn.115.111989 26511225 PMC4658459

[40] The CONSORT Statement. (2010). Available from: http://www.consort-statement.org/consort-2010

[41] Strobe Statement: Strengthening the Reporting of Observational Studies in Epidemiology. (2009). Available from: http://www.strobe-statement.org/index.php?id=strobe-home

